# Development and evaluation of an inhalable nanoemulsion system for enhancing NK cell function against osteosarcoma pulmonary metastases

**DOI:** 10.3389/fimmu.2026.1772375

**Published:** 2026-02-12

**Authors:** Sookyung Hwang, Paul D. Bates, Vasiliki Valkanioti, Chih-Chun Chang, Sandro Mecozzi, Christian M. Capitini

**Affiliations:** 1 Division of Pharmaceutical Sciences, University of Wisconsin School of Pharmacy, Madison, WI, United States; 2 Department of Pediatrics, University of Wisconsin School of Medicine and Public Health, Madison, WI, United States; 3 Department of Chemistry, University of Wisconsin-Madison, Madison, WI, United States; 4 Carbone Cancer Center, University of Wisconsin School of Medicine and Public Health, Madison, WI, United States

**Keywords:** inhalation, lung metastasis, nanoemulsion, NK cell, osteosarcoma, TGF-β, LILRB1, ILT-2

## Abstract

**Introduction:**

Osteosarcoma frequently metastasizes to the lungs, significantly reducing survival in pediatric and young adult patients, with current therapies having limited efficacy. This study aimed to develop an inhalable nanoemulsion formulation to enhance targeted pulmonary drug delivery and restore natural killer (NK) cell-mediated immunity against metastatic osteosarcoma.

**Methods:**

A nanoemulsion composed of medium-chain triglyceride (MCT) oil and Distearoyl-rac-glycerol-PEG_2000_ (DSG-PEG2000), yielding droplets consistently smaller than 200 nm, was designed with demonstrated stability (>550 days), biocompatibility, mucus penetration ability, minimal toxicity on respiratory epithelium, and efficient cellular uptake. To enhance NK cell function in the osteosarcoma tumor microenvironment, the nanoemulsion was loaded with SIS3, a SMAD3 inhibitor targeting immunosuppressive TGF-β signaling, and conjugated with a cysteine-modified form of the NK-cell killer immunoglobulin-like receptor (KIR) antagonist nonamer peptide VAPWNSDAL (VAP–DAC) to block inhibitory LILRB1/ILT-2 on NK-92 cells.

**Results:**

The resulting SIS3-VAP–DAC nanoemulsion maintained particle sizes below 162 nm, stability over one month, enhanced cytotoxicity of human NK-92 cells, and restoration of granzyme B secretion despite TGF-β suppression, as well as induced NKG2D ligand expression on murine osteosarcoma cells. Intranasal administration of the SIS3-VAP–DAC nanoemulsion effectively reduced pulmonary tumor burden in human osteosarcoma xenograft mouse models with no observable clinical toxicity.

**Discussion:**

This study establishes a novel inhalable nanoemulsion platform that significantly restores NK-cell functionality against pulmonary metastatic osteosarcoma.

## 1 Introduction

Osteosarcoma is the most prevalent primary bone cancer in children and young adults. Current standard approaches, primarily chemotherapy and surgical resection, have not markedly improved the 5-year survival rate in the past 50 years (1–3). Metastatic osteosarcoma, with a strong predilection for the lungs and other bones, has the poorest prognosis (4). Treatment options for pulmonary metastases remain limited to surgery and chemotherapy (5). Recent studies have emphasized the critical role of immune cells and their suppression by the tumor microenvironment in osteosarcoma progression (6). Natural killer (NK) cells are innate lymphocytes capable of eliminating tumor cells and have therapeutic potential in osteosarcoma (7). Clinical and preclinical findings indicate that pediatric osteosarcoma patients often exhibit decreased circulating NK cell counts (8). Additionally, transient NK cell depletion in athymic mouse models significantly enhances pulmonary metastasis of osteosarcoma (9). These findings emphasize the need for therapeutic strategies aimed at restoring NK cell activity in metastatic osteosarcoma (8). In order to restore NK cell function and reduce pulmonary tumor burden in osteosarcoma, we developed an inhalable nanoemulsion drug delivery system. This approach offers the potential for high local drug bioavailability, non-invasive administration, and potential for improved patient compliance (10, 11).

Nanomedicines are increasingly being developed to treat cancer (12), including osteosarcoma (13). In this report, an oil-in-water nanoemulsion was prepared using medium-chain triglyceride (MCT), a generally recognized as safe (GRAS)-listed compound by the Food and Drug Administration, as the oil core (14). DSG-PEG2000 was selected as a surfactant based on its safety profile and appropriate Hydrophilic-Lipophilic Balance (HLB) value (15). To restore NK cell function, the nanoemulsion was modified to express two agents: SIS3 and a modified form of the nonamer peptide VAPWNSDAL (VAP–DA) with a terminal cysteine, VAP–DAC.

SIS3 is a hydrophobic small molecule that selectively inhibits SMAD3 phosphorylation within the TGF-β signaling pathway, which is known to suppress NK cell function in the tumor microenvironment of various cancers, including osteosarcoma (16–18). In addition to its immunomodulatory effect, SIS3 inhibits osteoclastogenesis and tumor progression (19). Due to its poor solubility in aqueous medium and reliance on clinically unsuitable solvents like dimethyl sulfoxide (DMSO) or ethanol (20–22), SIS3 was dissolved in a safe oil medium at high concentration.

Killer immunoglobulin-like receptors (KIR) molecules expressed on human NK cells engage with human leukocyte antigen (HLA) on tumor cells to either activate or inhibit NK cell function. NK-92 cells are a commonly used interleukin (IL)-2 dependent NK cell line (23) that lacks expression of most activating and inhibitory KIRs except inhibitory KIR2DL4 (24, 25) as well as the KIR-related inhibitory receptor immunoglobulin-like transcript-2 (ILT-2), also known as LILRB1 (26). Potentially, immune checkpoint blockade of these inhibitory receptors could augment NK-92 function (27, 28). VAP–DA is a short synthetic nonamer peptide that functions as an antagonist for inhibitory KIR on NK cells by preventing their interaction with HLA-Cw*0102 and nascent peptides (29). KIRs that can bind HLA-Cw*0102 include KIR2DL2, KIR2DL3 and KIR2DS2, none of which are expressed on NK-92 cells (30). But the KIR-like molecule ILT-2 is expressed on NK-92 cells and binds HLA-C at low avidity (31), making NK-92 cells an ideal platform for testing if a peptide antagonist that blocks HLA-C binding augments NK function. A cysteine-modified form of VAP–DA, called VAP–DAC, was synthesized, purified, and conjugated to DSPE-PEG2000-Maleimide to incorporate VAP–DA onto a nanoemulsion system. A combined nanoemulsion SIS3-VAP–DAC was then tested alone and in combination with NK-92 cells to determine its effect on NK cell function *in vitro* and its ability to enhance potency of NK cells on osteosarcoma *in vivo*. This study successfully demonstrates the development of a stable, inhalable nanoemulsion capable of restoring NK cell function through blockade of TGF-β signaling and antagonizing an inhibitory receptor *in vitro* and *in vivo* through a metastatic osteosarcoma xenograft mouse model.

## 2 Materials and methods

### 2.1 Materials

Sterile normal saline (0.9% w/v sodium chloride, Cat #BXTAL4109) was obtained from Vyaire Medical, Inc. (Mississauga, ON). Distearoyl-rac-glycerol-PEG_2000_ (DSG-PEG2000, Cat #880152P) and Distearoyl-sn-glycero-3-phosphoethanolamine-PEG_2000_-maleimide (DSPE-PEG2000-maleimide Cat #880126P) were purchased from Avanti Polar Lipids, Inc. (Alabaster, AL). Distearoyl-sn-glycero-3-phosphoethanolamine-PEG_2000_ (DSPE-PEG2000 Cat #DSPE-020CN) was purchased from NOF America Corporation (North Broadway, NY). Labrafac Lipophile WL 1349 medium-chain triglyceride (MCT) oil and Plurol^®^ Diisostearique were gifted by Gattefossé (Paramus, NJ). Oxyma Pure (Cat #8.51086), trifluoroacetic acid (TFA) (Cat #T6508), thioanisole (Cat #T28002), porcine mucin Type II (Cat #M2378) and molecular-biology-grade agarose (Cat #A9539), Chitosan oligosaccharide lactate (Cat #523682), rink amide resin (Cat #855001) was obtained from Sigma-Aldrich (Milwaukee, WI).

N, N′-diisopropylcarbodiimide (DIC) (Cat #A19292.18), DiI (Cat #D3911), Nile red (Cat #N1142), tris(2-carboxyethyl) phosphine (TCEP) slurry gel (Cat #77712), and HEPES buffer (Cat #A14777.30) were from Thermo Fisher Scientific (Waltham, MA).

D-luciferin (Cat #222PS) was purchased from MediLumine (Montreal, QC). Airway Epithelial Cell Basal Medium and Bronchial Epithelial Cell Growth Kit, as well as Ham’s F-12K medium, were obtained from ATCC (Manassas, VA). DMEM and RPMI media, L-glutamine, MEM non-essential amino acids, and sodium pyruvate were supplied by Corning Inc. (Corning, NY). Fetal bovine serum (FBS) was sourced from GeminiBio (West Sacramento, CA), and penicillin/streptomycin and β-mercaptoethanol from Quality Biological (Gaithersburg, MD). Recombinant human (rh)IL-2 and rhIL-15 were obtained from the Biological Resources Branch (National Cancer Institute, Frederick, MD) and recombinant murine IL-15 was purchased from R&D Systems (Minneapolis, MN).

hSAEC primary small airway epithelial cells (Cat #PCS-301-010), NK-92 human NK cell lymphoma (Cat #CRL-2407), MG63 human osteosarcoma (Cat #CRL-1427), and K7M2 murine osteosarcoma (Cat #CRL-2836) cell lines were all obtained from ATCC (Manassas, VA). ATCC guidelines were followed for cell authentication using morphology monitoring, growth curve analysis, and testing for mycoplasma within 6 months of use.

### 2.2 Methods

#### 2.2.1 Mice

Twelve to sixteen-week-old male and female Balb/c (Charles River Laboratories, Wilmington, MA) or NOD scid gamma mice [NOD.Cg-*Prkdc^scid^ Il2rg^tm1Wjl^*/SzJ] (NSG) (Jackson Laboratories, Bar Harbor, ME) were used in this study. NSG mice were bred in an animal vivarium at the University of Wisconsin-Madison. All mice were housed and cared for following the Guide for the Care and Use of Laboratory Animals. All animal experiments were approved by the Institutional Animal Care and Use Committee (IACUC) under protocol M005915.

#### 2.2.2 Preparation of preliminary DSG-PEG2000 nanoemulsion

DSG-PEG2000 was solubilized in normal saline by sonicating at room temperature for more than 4 hours. The solution was then allowed to rest at room temperature overnight after sonication. Next, 2mL of Labrafac MCT, with or without hydrophobic dye, was added to the DSG-PEG2000 solution and vortexed briefly. Prior to emulsification, the homogenizer and microfluidizer were cleaned with 100% ethanol, 70% ethanol, 100% methanol, and Millipore Milli-Q water. The DSG-PEG2000/MCT mixture was homogenized at 21,500 rpm for 1 minute using a high-speed homogenizer (200 Homogenizer, VWR International, Randor, PA). After homogenization, the emulsion solution was further emulsified using a microfluidizer (Model M-110S, Microfluidics Corp., Newton, MA) at 5000 psi for 1 minute with an ice bath. The microfluidized emulsion was then filtered through a 0.45-μm, 25-mm PVDF syringe filter and stored at 4°C.

#### 2.2.3 Turbidity evaluation of DSG-PEG2000 with mucin

Mucin solutions (0.1% and 0.5% w/v) were prepared in sterile normal saline and stirred at 300 rpm using a magnetic stirring bar for more than 2 hours, until a slightly opaque solution with no sediment was obtained. Chitosan oligosaccharide lactate was dissolved in normal sterile saline at a concentration of 5 mg/mL. Full dissolution was observed without additional stirring. A 10 mM DSG-PEG2000 in sterile normal saline was prepared by sonication at room temperature for 4 hours. The sonicated solution was allowed to rest at room temperature overnight.

Chitosan oligosaccharide lactate or DSG-PEG2000 solution was mixed in an equal volume (1:1) with 0.5% mucin solution in a 20 mL scintillation vial. Sample for UV-spectrometry was collected every hour up to a 6-hour timepoint, then at a 24-hour timepoint. UV-Vis measurement was taken using Synergy H1 microplate reader (Biotek, Winooski, VT). Absorbance measurements were taken at 500 nm. The plate was shaken for 10 seconds before measurement, and the measurement was conducted at room temperature.

#### 2.2.4 Mucus penetration assay for DSG-PEG2000

Porcine Mucin Type II was used to make an artificial mucus layer that mimics the pulmonary mucus layer, as adopted from previously published studies by others (32, 33). Mucin Type II (5.5 g) was weighed into a glass beaker along with 11 mg of NaN3, and the total weight was adjusted to 110 g using 0.9% normal saline. The mixture was stirred with a magnetic bar at 300 rpm for 12 hours, producing a yellow, homogeneous solution with a slightly higher density than water.

For the 0.28% w/v agarose gel layer, 308 mg of agarose was dissolved in 110 mL of hot Millipore Milli-Q water using a microwave. Then, 2 mL of this hot agarose solution was transferred into scintillation vials with a plastic serological pipette, while the solution was kept hot on a low-heat magnetic stirrer. The agarose layers were allowed to harden at room temperature and subsequently stored at 4°C until use. The resulting layers were clear and free of air bubbles.

Two milliliters of 0.5% mucin solution were placed carefully at the top of the hardened agarose layer. No breach or damage to the agarose layers was observed upon mucin layer placement. The mucin layer was allowed to equilibrate on the agarose layer for 1 hour.

Nile-red-containing nanoemulsion (0.35 mM) was prepared as described before. Nile red solution with MCT (0.35 mM) (MCT-NR) was prepared for the control. Both nanoemulsion and NR-MCT were stored at room temperature for 2 hours before application. A 600-μL aliquot of each formulation was placed carefully at the top of the mucin layer, then the vials were incubated in the 37°C oven with lids closed. Physical force, such as vibration during transportation, was minimized during the procedure.

Samples for fluorescence measurement were collected every 2 hours up to a 6-hour timepoint. Upon sample collection, the mucin layer was removed carefully using a 2-mL plastic pipette. Then the agarose layer was washed with 2 mL of Milli-Q water (Millipore) three times, 20 seconds each time. Complete removal of the excess liquid was carried out using a syringe and needle. The agarose layer collected was then melted with microwave Proctor Silex 0.7 (Proctor Silex, Glen Allen, VA) at 700 Watts for 8–10 seconds per agarose layer. Mean absorbance of Nile red dye at 549–628 nm was measured using a Synergy H1 microplate reader (Biotek, Winooski, VT).

#### 2.2.5 *In vitro* toxicity assessment of nanoemulsion on hSAEC cells

A human primary small airway epithelial cell line (hSAEC) was used to evaluate the impact of the nanoemulsion on cell viability. Cells were cultured in Airway Epithelial Cell Basal Medium (ATCC, Manassas, VA) supplemented with a Bronchial Epithelial Cell Growth Kit (ATCC). Cells were plated in a 96-well plate at a density of 5,000 cells/well and incubated at 37°C for 24 hours. Following incubation, cells were treated with blank nanoemulsion at concentrations of 20, 40, 60, 80, and 100% (v/v) and further incubated at 37°C for 24 hours. Afterward, CellTiter-Blue reagent (10 μL; Promega) was added to each well, and the plate was incubated for an additional 3 h at 37°C. Fluorescence intensity (excitation: 560 nm, emission: 590 nm) was measured using a Synergy H1 microplate reader (BioTek). Cell viability for each treatment was calculated relative to untreated control wells (n = 5).

#### 2.2.6 VAP–DAC peptide synthesis, purification, and conjugation

A C-terminal cysteine was added to the KIR-antagonist nonamer peptide VAPWNSDAL to allow site-specific thiol-maleimide conjugation to the DSPE-PEG2000- Maleimide. The modified peptide is denoted as VAP–DAC.

VAP–DAC peptide was synthesized via solid-phase peptide synthesis (SPPS) using a Liberty Blue 2.0 peptide synthesizer (CEM Corporation, Matthews, NC). Rink amide resin (0.156 g; 0.4 mmol/g loading) was employed in a 30-mL reaction vessel. Amino acids and coupling reagents were dissolved in dimethylformamide (DMF); 10% piperidine in DMF was used for deprotection, and 1 M solutions of Oxyma and DIC in DMF were used for coupling. Coupling and deprotection were automated per standard protocols, with residues capped using a 3% acetic anhydride solution in DMF. Post-synthesis, the resin-bound peptide was washed thoroughly with DMF and dichloromethane (DCM). The peptide was cleaved from the resin using a cocktail of TFA, thioanisole, triisopropylsilane (TIS), and water (85:5:5:5 v/v/v/v) for 2 hours, precipitated in chilled diethyl ether, filtered, washed, and lyophilized overnight at -50°C (~150 μPsi).

For purification, approximately 10 mg of freeze-dried peptide was dissolved (10 mg/mL in 1:1 acetonitrile (ACN):water). Semi-preparative HPLC was performed (Agilent 1260 Infinity II) using a ZORBAX StableBond C18 column (9.4 x 250 mm, 5 µm). The mobile phases comprised A: 1% TFA in water and B: 1% TFA in ACN, running a linear gradient from 90% A to 40% A over 50 min, held at 40% A for 5 min, then further gradient to 20% A over 10 min, before returning to initial conditions. Detection wavelengths were 214 nm, 230 nm, and 254 nm at a flow rate of 2 mL/min and column temperature of 25°C. Peptide identity and purity were confirmed by MALDI-TOF mass spectrometry (Autoflex III, Bruker Daltonics, Billerica, MA).

For conjugation, 10 mM HEPES buffer (pH 7.0) was prepared. Pure peptide (2.02 mg) dissolved in HEPES buffer was reduced using TCEP slurry gel (0.5 mL), incubated for 1 hour at room temperature (RT), centrifuged, and supernatant collected under inert gas. DSPE-PEG2000-Maleimide (17.77 mg, 1:3 molar ratio) was dissolved in HEPES buffer, added dropwise to the reduced peptide solution, and stirred for 2 hours at RT. The reaction was quenched with N-acetyl-L-cysteine (10 µmol). The mixture was dialyzed overnight (2000 MWCO dialysis cassette), lyophilized (-35 °C, 150 µPsi), and stored at -80 °C. Conjugation efficiency was validated by MALDI-TOF mass spectrometry.

#### 2.2.7 Preparation of SIS3 containing VAP–DAC nanoemulsions

Oil-in-water nanoemulsions were prepared using high-speed homogenization and microfluidization methods. Pharmaceutical-grade medium-chain triglyceride (MCT; Labrafac Lipophile WL 1349) was selected as the oil phase. SIS3 was dissolved in MCT oil via sonication at 60-65°C overnight, with its concentration confirmed by HPLC. The surfactant system included DSG-PEG2000 at 9.95 mM and DSPE-PEG2000 at 0.05 mM (NE1 and NE2), or VAP–DAC-maleimide-DSPE-PEG2000 at 0.05 mM (NE3 and NE4) (**Supplementary Table 1**). The aqueous phase consisted of either 1x phosphate-buffered saline (PBS) or sterile normal saline (0.9% w/w sodium chloride). Emulsification involved high-speed homogenization (21,500 rpm, 1 min) followed by microfluidization (7000 psi, 1 min), with subsequent filtration through a 0.45 μm polyvinylidene fluoride (PVDF) syringe filter. Nanoemulsions NE5-NE7 (**Supplementary Table 1**) followed a similar synthesis protocol but utilized an oil phase composed of MCT: Plurol^®^ Diisostearique (9:1, v/v) to aid dissolution of SIS3 in higher concentration. SIS3 concentration in this modified oil phase was similarly measured by HPLC.

#### 2.2.8 Dynamic light scattering

Preliminary nanoemulsion formulations were diluted with a dilution factor of 600 in Millipore Milli-Q water. Dynamic light scattering was conducted using NICOMP 380 ZLS (Particle Sizing Systems, Santa Barbara, CA). Particle size analysis run was conducted for 5 minutes at room temperature. Polystyrene cuvette was used, and measurements were repeated three times. Gaussian analysis was applied to the data, with the results expressed as intensity-weighted average diameters.

Nanoemulsion formulations with SIS3 and/or VAP–DAC were diluted with a dilution factor of 100 in Millipore Milli-Q water. Dynamic light scattering was conducted using Zetasizer Nano ZS (Malvern Panalytical Inc., Westborough, MA). Particle size analysis and zeta potential analysis runs were conducted for 5 minutes each at room temperature. DTS1070 cuvettes (Malvern Panalytical Inc.) were used, and measurements were repeated three times. Gaussian analysis was applied to the data, with the results expressed as intensity-weighted average diameters. All nanoemulsions were stored at 4°C.

#### 2.2.9 *In vitro* IncuCyte cytotoxicity assay

Green fluorescent protein (GFP)-expressing MG63 human osteosarcoma cells and NK-92 human NK cells were co-cultured in an IncuCyte (Sartorius, Ann Arbor, MI) live cell imaging cytotoxicity assay at an effector-to-target (E:T) ratio of 5:1. Prior to the assay, NK-92 cells were irradiated with gamma rays (100 Gy) to inhibit proliferation during co-incubation and maintained with IL-2 at 20 IU/mL (34). Nanoemulsions were prepared by diluting with DMEM (Corning Inc., Corning, NY) cell culture media supplemented with FBS 10%, penicillin/streptomycin 1%, L-glutamine 1%, HEPES 1%, MEM 1%, sodium pyruvate 1%, 2 nM beta-mercaptoethanol 0.1%, and IL-2–100 IU/mL. NK-92 cells were expanded in similar culture media. For MG63 cells, RPMI (Corning) cell media with the same supplements were used for expansion. Nanoemulsions were diluted to a final concentration of 50% (v/v) with cell media, with all experimental wells containing the respective nanoemulsions.

MG63 and NK-92 cells were separately incubated with their corresponding nanoemulsion preparations for 8 hours, with thorough vortexing (~10 sec) of nanoemulsions both before and after dilution. Following incubation, cells were centrifuged and washed once with warm PBS (Corning). Subsequently, cell counts were determined, and cells were seeded into 96-well plates, maintaining an adjusted E:T ratio of 5:1. Each well was supplemented with recombinant TGF-β1 (Thermo Fisher Scientific, Waltham, MA) at a concentration of 5 ng/mL.

Fluorescent images were captured using the IncuCyte imaging system (Sartorius) every 2 hours over a total duration of 48 hours, enabling continuous monitoring of GFP^+^ MG63 cell viability. The experimental groups tested included (1): Negative control (PBS only), (2) NE1 (blank nanoemulsion), (3) NE2 (SIS3-only nanoemulsion), (4) NE3 (VAP–DAC-peptide-only nanoemulsion), and (5) NE4 (SIS3-VAP–DAC nanoemulsion).

#### 2.2.10 Isolation and expansion of primary murine NK cells

Bone marrow was harvested from tibias and fibulas from Balb/c mice, processed in RPMI by mortar and pestle to a single-cell suspension, and subjected to red blood cell lysis using ACK lysis buffer (Lonza, Walkersville, MD). NK cells were isolated via magnetic cell separation with an NK cell isolation kit on an autoMACS Pro (Miltenyi Biotec, San Jose, CA). A total of 2 × 10^6^ NK cells were expanded for 3 days in RPMI 1640 medium (Corning Life Sciences, Durham, NC) supplemented with 10% FBS (GeminiBio, West Sacramento, CA); penicillin (100 U/mL) and streptomycin (100 µg/mL) (Quality Biological, Gaithersburg, MD); 94 µM 2-mercaptoethanol (Gibco, Carlsbad, CA); 10 mM MEM non-essential amino acids (100×) (Corning Life Sciences, Durham, NC); 12.9 mM HEPES buffer (Corning Life Sciences, Durham, NC); and 1 mM sodium pyruvate (Corning Life Sciences, Durham, NC). Recombinant murine IL-15 (1 µg/mL) was added at initiation of the culture. The culture medium was replaced with fresh, fully supplemented medium every 2–3 days.

#### 2.2.11 Flow cytometric analysis of NKG2D ligand induction

After 3 days of culture, murine NK cells were counted and analyzed by flow cytometry following co-incubation with K7M2 murine osteosarcoma cells (ATCC, Manassas, VA) at a 1:1 E:T ratio and nanoemulsion. A total of 5×10^5^ cells were cultured with each nanoemulsion (NE1–NE4) at 50% (v/v) for 12 hours. Cells were then washed in FACS buffer containing PBS with 0.2% FBS and 0.1% sodium azide (Sigma Aldrich) and stained with 1 μL anti-RAE-1 antibody (BioLegend, San Diego, CA) and 1 μL anti-NKp46 antibody (BioLegend) at 4°C for 30 min. Cells were washed in PBS at 300 g for 7 minutes and resuspended in 200 μL FACS buffer. Data acquisition was performed on an Attune NxT flow cytometer (Thermo Fisher Scientific, Waltham, MA), and analysis was carried out using FlowJo v10 software (FlowJo LLC, Ashland, OR).

#### 2.2.12 Human granzyme B ELISA

The secretion of human granzyme B was measured using a human granzyme B ELISA kit from Proteintech (Proteintech, Rosemont, IL, Cat #KE00121). NK-92 cells were cultured in 25-mL flasks at a density of 6 × 10^5^ cells per flask in DMEM cell media supplemented as described above. Cells were assigned to four experimental groups: (1) negative control (cell media only), (2) IL-15 only, (3) IL-15 + TGF-β, and (4) IL-15 + TGF-β + NE7. Treatments, including rhIL-15 (20 ng/mL), TGF-β (5 ng/mL), and NE7 (50% v/v with cell media), were added directly to the cell media as specified for each group. Cells were incubated for 48 hours, after which supernatants were collected and diluted at a 1:160 ratio prior to ELISA analysis.

#### 2.2.13 *In vivo* LAGO bioluminescence imaging of human osteosarcoma pulmonary metastases

Twenty NSG mice were injected intravenously via the tail vein with MG63-luciferase (luc) cells (3.5 million cells per mouse). After 28 days, pulmonary metastasis of MG63-luc cancer cells was confirmed using the LAGO Imaging System (Spectral Instruments Imaging, Tucson, AZ) after intraperitoneal injection of the bioluminescent reporter, D-Luciferin (MediLumine Inc., Montreal, QC). All 20 mice were divided into four experimental groups: untreated saline control (5 females), NE5 blank nanoemulsion group (5 males), NE6 SIS3 nanoemulsion group (5 females), and NE7 SIS3-VAP–DAC nanoemulsion group (5 males). The control group received 20 μL saline, whereas the NE5, NE6, and NE7 groups each received 20 μL of respective nanoemulsions. Treatments were administered intranasally three times per week. Additionally, all mice received weekly tail-vein injections of 1 million irradiated NK-92 cells supplemented with 100 IU IL-2. Further, an intraperitoneal injection of IL-2 (20 IU per mouse) was administered weekly to promote NK survival *in vivo*.

Imaging was performed weekly throughout a 3-week treatment period. Osteosarcoma tumor burden in mice was assessed by bioluminescent imaging using the LAGO X imaging system following intraperitoneal injection of D-luciferin (3mg per mouse, 100 μL per mouse, 30 mg/mL). Images were taken after 15–20 minutes from D-luciferin injection. Regions of interest (ROIs) were drawn over the chest area and quantified using Aura imaging software (Spectral Instruments Imaging, Tucson, AZ), with identical ROI shape and size applied across all samples to ensure consistency. Mice were weighed twice weekly throughout the dosing period, and their clinical condition was scored using the following criteria (**Supplementary Table 2**). Body weight change was expressed as a percentage change from baseline for each animal.

#### 2.2.14 Statistical analysis

All statistical analyses were performed using R (The R Foundation for Statistical Computing, Vienna, Austria) and RStudio (Posit, Boston, MA, USA). For *in vitro* experiments, group comparisons were analyzed using one-way ANOVA followed by Tukey’s HSD *post hoc* test. Data from IncuCyte cytotoxicity assays were assessed using the Kruskal-Wallis test with Benjamini–Hochberg (BH) adjusted Dunn’s multiple comparison test. For *in vivo* bioluminescence imaging and tumor burden analyses, the Kruskal-Wallis test followed by Dunn’s *post hoc* test with Bonferroni correction was applied. Data visualization was performed using the ggplot2 package in R.

## 3 Results

### 3.1 Preparation of preliminary nanoemulsion and physicochemical characterization

**Figure 1:**
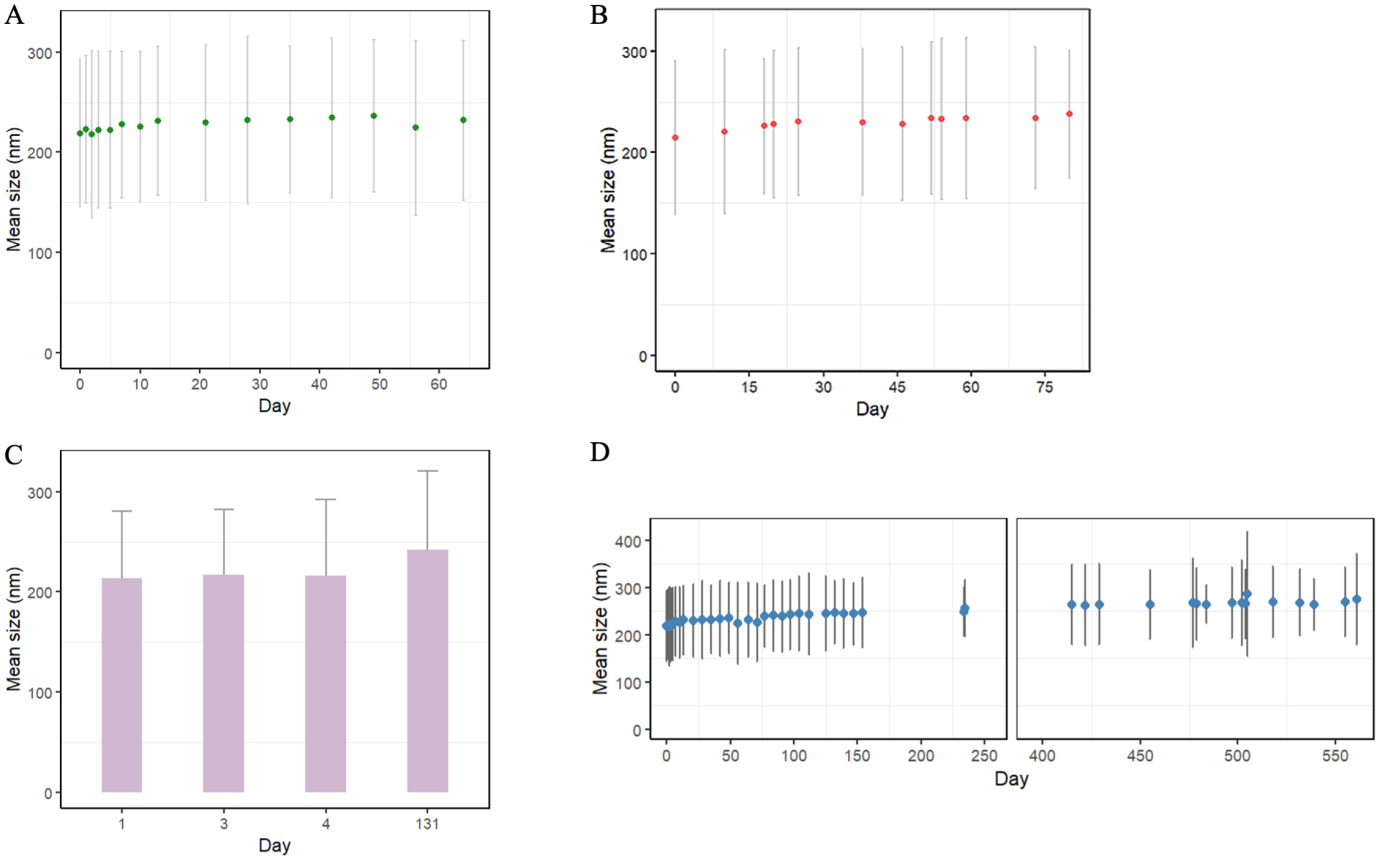
Change in mean particle size of nanoemulsion over time as measured by dynamic light scattering. Error bars represent standard deviation. **(A)** Change in mean particle size of blank nanoemulsion over a 61-day period, **(B)** Change in mean particle size of Nile red-containing nanoemulsion (0.35 mM), **(C)** Change in mean particle size of DiI-containing nanoemulsion (629 μM) over time, **(D)** Change in mean particle size of blank nanoemulsion over a 1-year period as measured by dynamic light scattering.

In order to activate NK cells within the pulmonary osteosarcoma tumor microenvironment, an intranasal nanoemulsion was prepared as a surfactant that had a stable shelf life. After preparation, the mean particle size of the nanoemulsion droplets was measured by dynamic light scattering. When freshly prepared, nanoemulsions were within the lower end of the 200 nm size range, and this value gradually increased over time, insignificantly. The mean particle size of blank nanoemulsion was 206.2 ± 70 nm when freshly prepared, then increased up to 223.8 ± 53 nm over a 61-day period (**Figure 1A**). The mean particle size of Nile red-containing (0.35 mM) nanoemulsion was 214.7 ± 77 nm, then increased to 237.6 ± 63 nm over an 80-day period (**Figure 1B**). Similarly, the mean particle size of DiI-containing (629 μM) nanoemulsion was 213.8 ± 66 nm when freshly prepared, then increased gradually to 241.8 ± 79 nm over a 131-day period (**Figure 1C**). Long-term physical stability of the blank nanoemulsion was observed as particle size did not increase significantly over a 1-year period, with a size range within 200 nm (**Figure 1D**). No phase separation or other visual instability was observed in all nanoemulsions.

### 3.2 Turbidity evaluation of DSG-PEG2000 with mucin

The mucus layer is a viscoelastic aqueous mixture that forms a barrier lining the upper airway tract (35). The mucus layer provides an effective mesh-like filter (36), which hinders the effectiveness of the drug delivery by nanocarriers. The main component of the mucus layer is mucin, a large, heavily glycosylated polymer that provides structural integrity and protection to human airway surfaces (37). Mucins electrostatically interact with cationic polymers such as chitosan, causing aggregation (38). Polymers with mucoadhesive properties will entangle with mucin glycoprotein in the mucus layer, leading to an increase in turbidity of the solution due to this slight aggregation (39–41). PEG-based drug delivery systems are often known to be mucopenetrating with a high diffusion coefficient when lower-molecular-weight PEGs are employed for coating (42).

**Figure 2:**
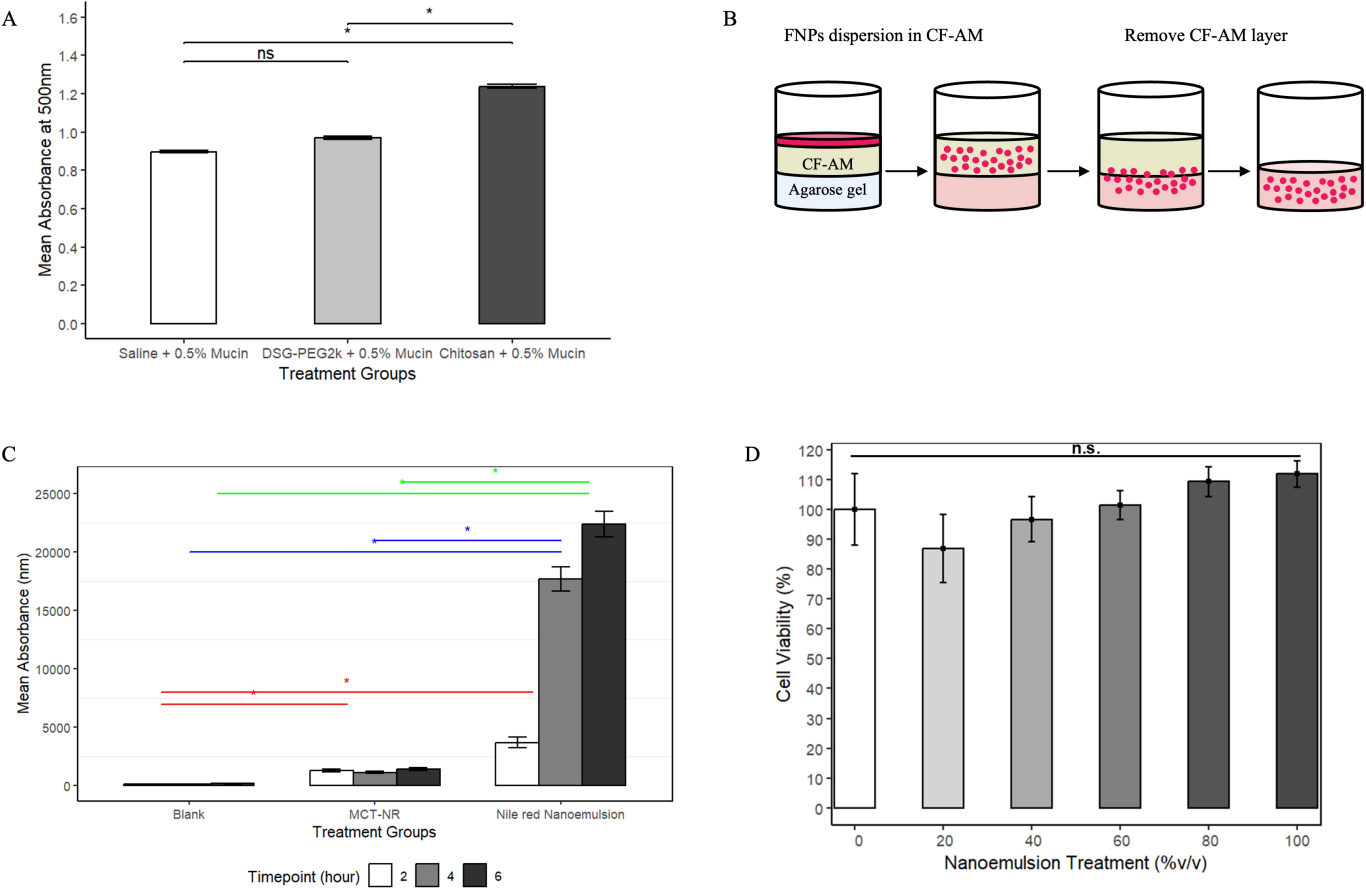
DSG-PEG2000 turbidity assessment, mucus penetration potential of DSG-PEG2000, and toxicity analysis of DSG-PEG2000 on human respiratory epithelial cells. **(A)** Turbidity measurement of normal saline, DSG-PEG2000, and chitosan oligosaccharide lactate solutions added to 0.5% porcine mucin II solution, with significance indicated. Error bars represent the standard error of the mean values (* indicates significant difference (p < 0.01), n=3). **(B)** Visual schematic of artificial mucus layer penetration study (figure created with PowerPoint). **(C)** Mean absorbance of Nile red in the agarose layer due to mucopenetration. The red line indicates significance between the timepoint 2 hr group. Blue line indicates significance between the timepoint 4 hr group. The green line indicates significance between the timepoint 6 hr group. Error bars represent standard deviation (* indicates significant difference (p < 0.01)). **(D)** Viability of hSAEC cells following incubation with blank nanoemulsion at concentrations of 20, 40, 60, 80, and 100% (v/v).

*In vitro* turbidity evaluation and mucus layer penetration studies were carried out to predict the interaction of the nanoemulsion system with the mucus layer after inhalation. Sterile normal saline was used as a negative control, whereas chitosan oligosaccharide lactate was used as a positive control. Two different concentrations of porcine Mucin II solution in normal saline were added to the same volume of 10 mM DSG-PEG2000 or ~1 mM chitosan oligosaccharide. UV-visible spectrometry was conducted after addition to detect aggregation and therefore to determine the mucoadhesiveness of the DSG-PEG2000 polymer. Results indicated no significant difference between DSG-PEG2000 treatment groups and mucin-alone groups prepared with normal saline (**Figure 2A**). However, turbidity from chitosan groups containing 0.5% mucin was significantly higher than both other groups (p < 0.01) (**Figure 2A**). No significant differences were observed among groups containing 0.1% mucin.

### 3.3 Mucus penetration ability of DSG-PEG2000

Neutral-charged, sufficiently hydrophilic, and nanoscale carriers are required to evaluate potential airway toxicity upon inhalation (32). Given that DSG-PEG2000 is known to be non-mucoadhesive, confirming its mucopenetrating capability is essential to ensure that the resulting nanoemulsion effectively penetrates mucus, thus enhancing drug delivery upon inhalation.

A hydrophobic dye (Nile red, 0.35 mM) was used to evaluate potential airway toxicity upon inhalation (**Figure 2B**). Briefly, the agarose gel layer in a vial represents the airway epithelium under the mucus layer. The artificial mucus layer made from porcine mucin II represents the mucus layer lining at upper airway tract. Nile red dissolved in MCT oil (MCT-NR) (0.35 mM) was selected as the positive control. No formulation was applied to the negative control. The resulting mean absorbance values indicate that the Nile red nanoemulsion successfully delivered its hydrophobic cargo, Nile red, into the agarose layer through the mucus barrier. The Nile red nanoemulsion demonstrated significantly greater Nile red delivery at 4- and 6-hour time points compared to the positive control group. In contrast, the MCT-NR positive control group did not exhibit significant delivery of Nile red into the agarose layer after the 2-hour time point (**Figure 2C**).

### 3.4 *In vitro* toxicity assessment of nanoemulsion on primary small airway epithelial cells

Human primary small airway epithelial cells (hSAEC) were exposed to blank nanoemulsion diluted in 1× PBS at concentrations ranging from 20% to 100% (v/v) to evaluate potential airway toxicity upon inhalation. After 24 hours of incubation, cell viability was assessed using a CellTiter-Blue assay. Overall, the blank nanoemulsion did not significantly reduce hSAEC cell viability at any tested concentration, confirming its biosafety towards airway epithelial cells (**Figure 2D**). No statistically significant differences were observed between any treatment group and the untreated control.

### 3.5 VAP–DAC peptide synthesis, purification, and conjugation

**Figure 3:**
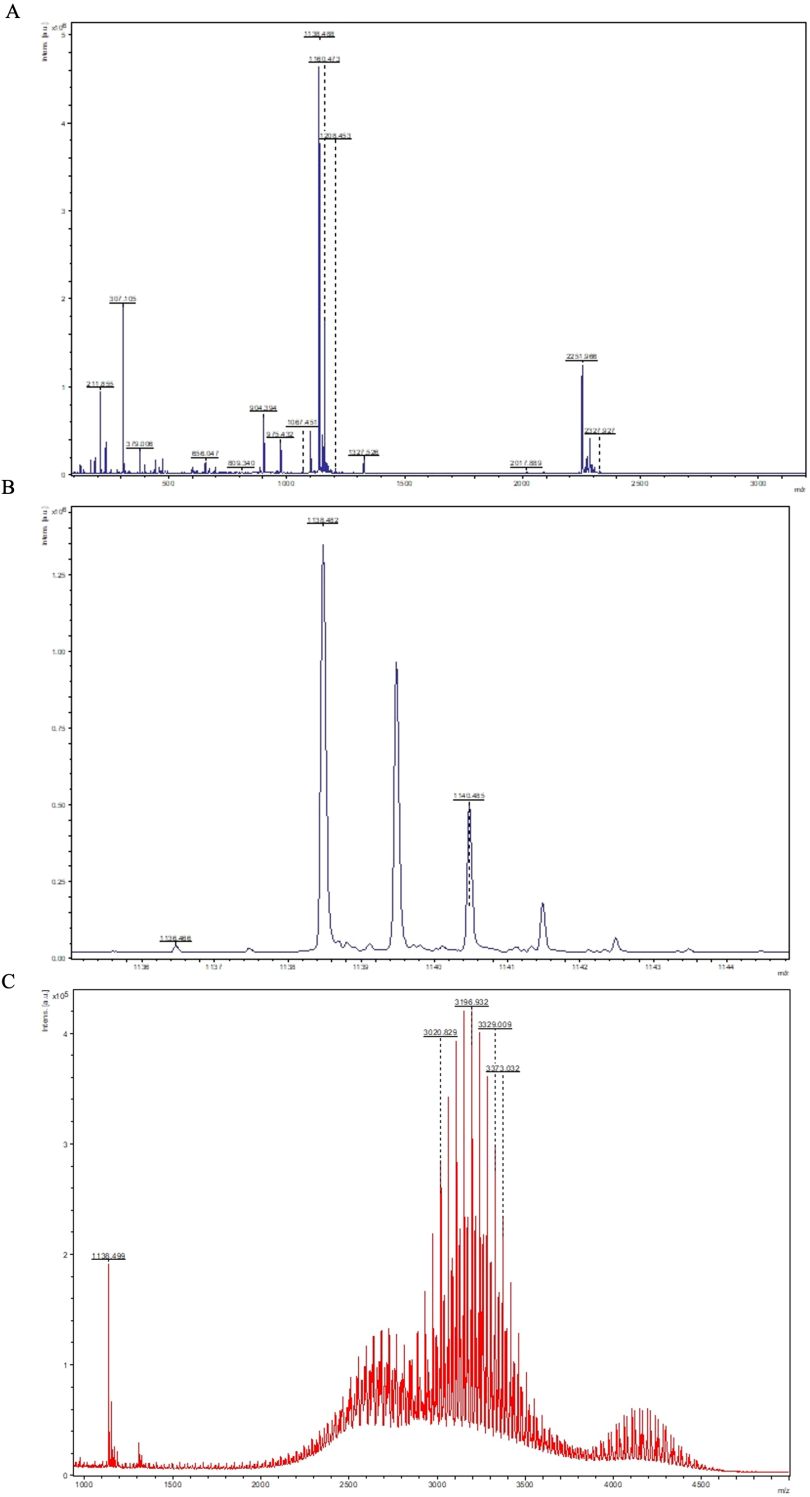
Confirmation of expression of VAP–DAC peptide on the DSG-PEG2000 nanoemulsion. MALDI-TOF mass spectrometry analysis of the eluate collected at 28-minute retention time. **(A)** The overall spectrum displays two major peaks corresponding to the monomeric peptide (m/z ~1138.49) and the disulfide-linked dimeric form (m/z ~2251.96). **(B)** A magnified view of the spectrum confirms the presence of the monomeric VAP–DAC peptide with an observed m/z of approximately 1138.49. **(C)** MALDI-TOF mass spectrometry analysis of purified VAP–DAC-DSPE-PEG2000-Maleimide conjugate.

VAP–DAC peptide was synthesized as a component of the nanoemulsion surface to prevent HLA-C on tumor cells from inactivating NK cells. MALDI-TOF mass spectrometry confirmed the presence of both monomeric peptide (m/z ~1138.49) (**Figure 3A**) and its dimeric disulfide-linked form (m/z ~2251.96) from the elute collected at a 28-minute retention time (**Figure 3B**). Subsequent purification steps using HPLC improved purity, with the final purified peptide product achieving an approximate cumulative total of 10 mg. Successful conjugation of VAP–DAC to DSPE-PEG2000-Maleimide was confirmed by MALDI-TOF mass spectrometry (**Figure 3C**). The conjugation was based on thiol–maleimide click chemistry, in which the thiol group of the cysteine residue selectively reacts with the maleimide group under mild aqueous conditions to form a stable thioether bond. Owing to the inherent polydispersity of the PEG polymer, the resulting spectrum displayed a broad m/z distribution. Nevertheless, the appearance of a distinct polymer–peptide conjugate peak, absent in the spectrum of purified VAP–DAC alone, confirmed successful conjugation.

### 3.6 Physicochemical characteristics and stability profile of NK cell activating nanoemulsions

Osteosarcoma uses contact-dependent and contact-independent ways to inactivate NK cells. Expression of HLA-C and nascent peptides engages inhibitory KIRs and KIR-like molecules like ILT-2 on NK cells, inactivating their function in a contact-dependent manner. Introducing a synthetic nonamer peptide like VAP–DAC could interfere with KIR or ILT-2 engagement with HLA-C and restore NK function. TGF-β production by tumor cells generates an immunosuppressive tumor microenvironment that inhibits NK cell function in a contact-independent manner. Incorporating SIS3 into the nanoemulsion would prevent inhibition of NK cells from TGF-β present in the osteosarcoma tumor microenvironment by blocking SMAD3, which is a signaling molecule downstream of the TGF-β receptor on NK cells.

**Figure 4:**
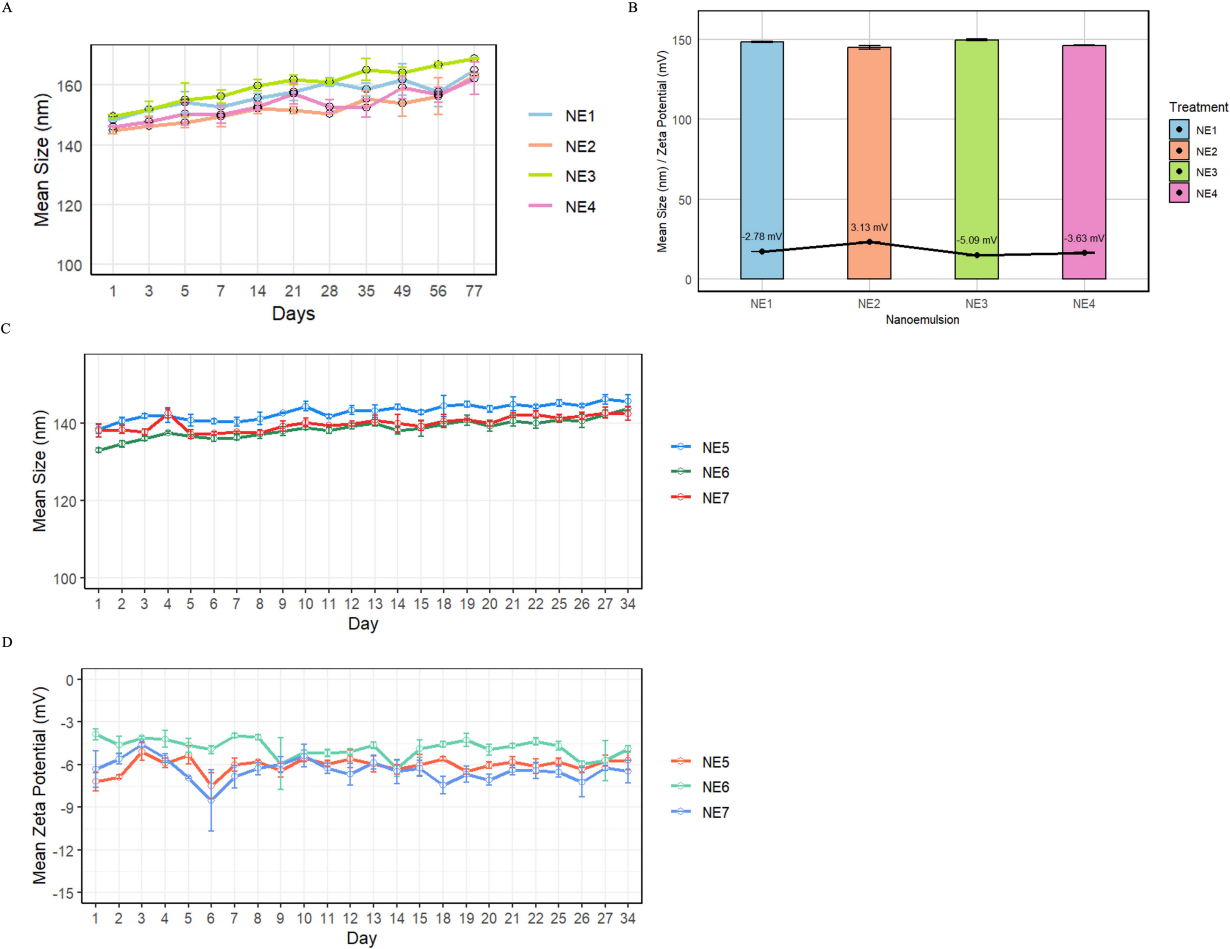
Measurement of nanoemulsion size after incorporation of SIS3 and VAP–DAC. Change in mean particle size and zeta potential of nanoemulsion NE1-NE7 over time as measured by dynamic light scattering. Error bars represent standard deviation. **(A)** Change in mean particle size of NE1-NE4 nanoemulsions over time as measured by dynamic light scattering. **(B)** Mean particle size and zeta potential of NE1-NE4 nanoemulsions measured on the day of preparation. **(C)** Change in mean particle size of NE5-NE7 nanoemulsions over time as measured by dynamic light scattering. **(D)** Mean zeta potential of NE5-NE7 nanoemulsions measured over time.

Four different nanoemulsion formulations were tested, examining a blank nanoemulsion (NE1), a SIS3-containing nanoemulsion (NE2), a VAP–DAC-containing nanoemulsion (NE3), and a dual SIS3-VAP–DAC nanoemulsion (NE4). The mean particle size of the NE droplets was measured by dynamic light scattering. NE1-NE4 formulations all exhibited initial mean diameters below 150 nm, which gradually increased to approximately 165 nm over a 77-day period. Specifically, NE1 increased from 148.36 nm to 165.06 nm; NE2 from 144.93 nm to 163.46 nm; NE3 from 149.66 nm to 168.90 nm; and NE4 from 146.20 nm to 162.33 nm (**Figure 4A**). When freshly prepared, all four nanoemulsions displayed near-neutral zeta potentials (-5.09 mV to +3.13 mV), indicating minimal surface charge (**Figure 4B**).

Because SIS3 is a highly insoluble hydrophobic compound, we further optimized the nanoemulsion formulation to improve SIS3 solubility and increase drug loading. Therefore, for the second series of nanoemulsions, an optimized co-surfactant system composed of MCT and Plurol^®^ Diisostearique, along with a higher SIS3 concentration, was used to achieve higher SIS3 loading. As a result, SIS3 solubility in the oil phase was enhanced, and concentrations of approximately 240 μM were achieved. A higher pressure (7000 psi) for microfluidization was also applied to improve droplet uniformity and nanoemulsion stability. Increasing microfluidization pressure to 7000 psi reduced droplet size to approximately 140 nm, maintaining stability over 34 days with near-neutral zeta potentials (-3.9 to -7.2 mV). The resulting formulations demonstrated a minimal droplet size increase (≤ 20 nm) over a 77-day period, and HPLC analysis confirmed successful loading of SIS3 at 124.9 µM in the MCT oil phase following sonication. NE5 (blank nanoemulsion) grew from 138.46 nm to 145.66 nm over 34 days; NE6 (SIS3 nanoemulsion) from 132.96 nm to 143.73 nm over 34 days; and NE7 (SIS3-VAP–DAC nanoemulsion) from 138.10 nm to 142.46 nm over 34 days (**Figure 4C**). Mean particle sizes of NE5-NE7 were similarly consistent at the upper end of the 130 nm range and showed only minor increases over 34 days. No phase separation or other visual instability was observed in any formulation. Mean zeta potential for NE5-NE7 was monitored over 34 days using a Malvern Zetasizer. All three maintained slightly negative surface charges throughout storage. NE5 shifted from -7.19 mV to -5.71 mV, NE6 from -3.86 mV to -4.89 mV, and NE7 from -6.31 mV to -6.49 mV (**Figure 4D**). These data confirm that all nanoemulsions remained colloidally stable, exhibiting only minor surface-charge variations over one month of storage.

### 3.7 *In vitro* IncuCyte cytotoxicity with SIS3-VAP–DAC nanoemulsion

**Figure 5:**
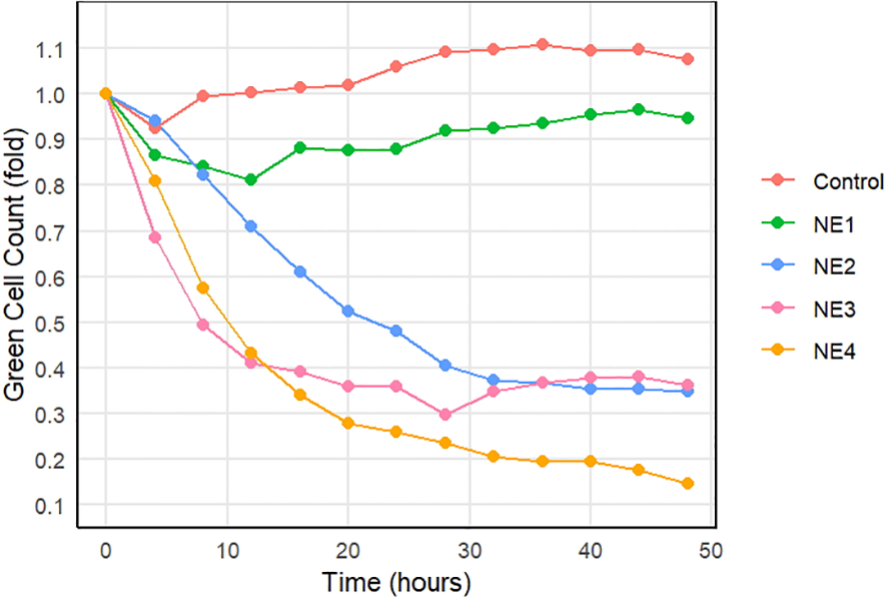
SIS3-VAP–DAC nanoemulsion enhances *in vitro* NK-92 cell-mediated cytotoxicity of osteosarcoma in the presence of TGF-β. Fold-change in MG63-GFP cell count measured by IncuCyte over 48 h following co-incubation with irradiated NK-92 cells (E:T 5:1) in media supplemented with 5 ng/mL TGF-β and 50% (v/v) NE1 (blank), NE2 (SIS3), NE3 (VAP–DAC), or NE4 (SIS3-VAP–DAC) nanoemulsions.

Green cell counts of MG63 osteosarcoma cells were measured after co-culture with NK-92 cells and a nanoemulsion using the IncuCyte imaging system over a 48-hour period, with reduced green counts indicating NK cell cytotoxicity of the tumor cells. Inclusion of either a VAP–DAC-containing nanoemulsion (NE3) or a dual SIS3-VAP–DAC nanoemulsion (NE4) significantly enhanced NK cytotoxicity as compared to the control, NE1, or NE2 groups (p < 0.001) (**Figure 5**). Of note, both NE3 and NE4 nanoemulsions enhanced NK-92 cell cytotoxicity against MG63 cells in the presence of TGF-β.

### 3.8 Flow cytometry: murine NK cells and K7M2 cells

Flow cytometry showed that VAP–DAC-containing nanoemulsions resulted in higher RAE-1 expression on K7M2 murine osteosarcoma cells when co-incubated with NK cells. RAE-1, a ligand for the NK-cell activating receptor NKG2D (43), is known to exhibit reduced expression on cancer cells within tumor microenvironments rich in immunosuppressive mediators, including TGF-β (44). Nanoemulsions containing the VAP–DAC peptide (NE3 and NE4) significantly increased RAE-1 expression on K7M2 cells (33.3% and 26.3%, respectively), compared to peptide-free formulations (2.15% and 8.48%, respectively). These findings indicate that VAP–DAC-functionalized nanoemulsions have the potential to enhance NKG2D-dependent immune surveillance (**Supplementary Figure 1**).

### 3.9 Granzyme B production by NK-92 cells

**Figure 6:**
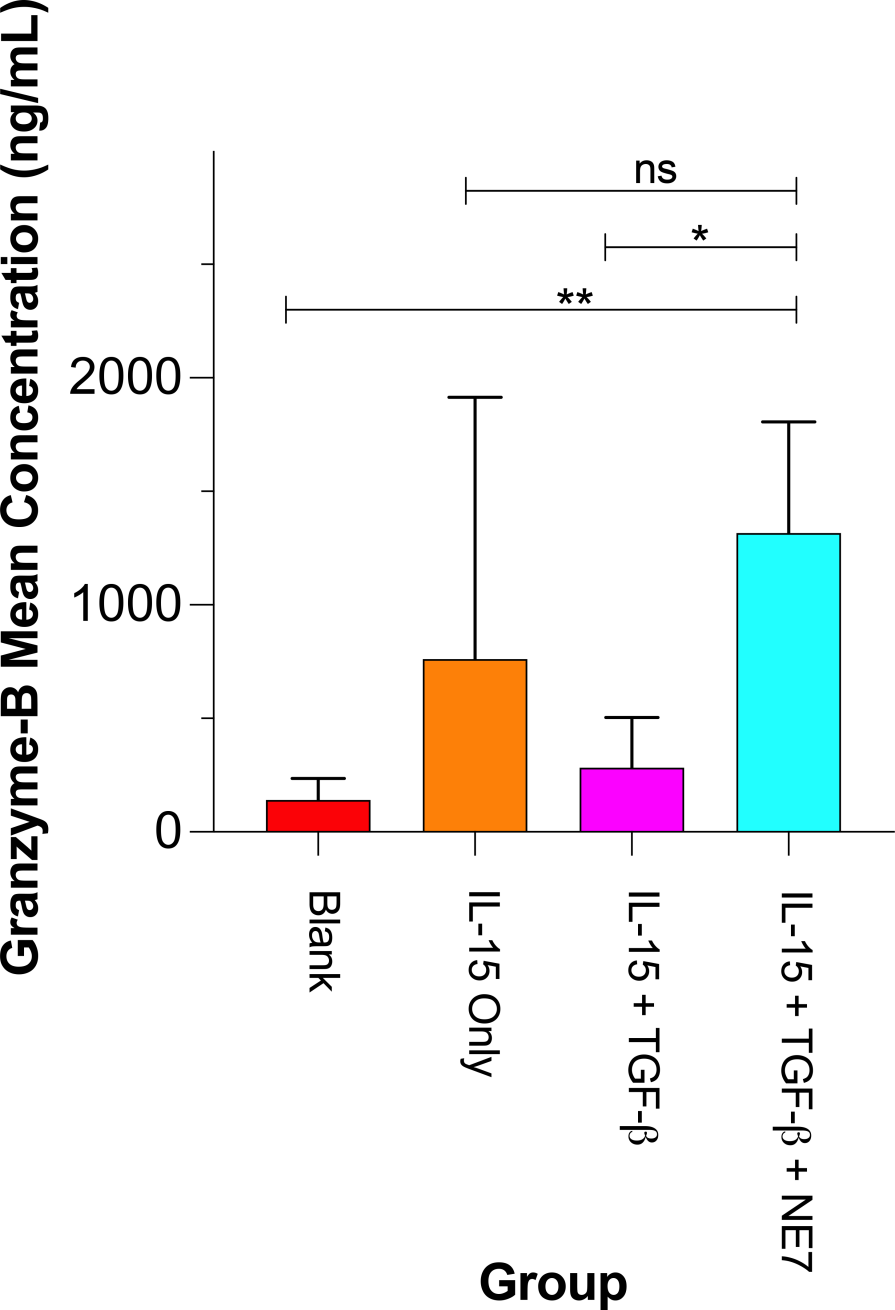
SIS3-VAP–DAC nanoemulsion enhances granzyme B production by IL-15 activated human NK-92 cells even in the presence of TGF-β. Bar graph of human granzyme B concentration (ng/mL) for treatment groups: Blank (PBS) control; IL-15 (20 ng/mL); IL-15 (20 ng/mL) + TGF-β (5 ng/mL); and IL-15 + TGF-β + 50% (v/v) NE7 (SIS3-VAP–DAC) nanoemulsion. (**p ≤ 0.01, *p < 0.05, n.s: p ≥ 0.05, n = 3~6).

Granzyme B is a key component of cytotoxic granules secreted by NK cells to eliminate cancer cells. However, tumor microenvironments enriched with TGF-β are known to suppress NK cell function by reducing the secretion of granzyme B and perforin (45). In this study, the NE7 nanoemulsion (containing both SIS3 and the VAP–DAC peptide) was evaluated for its potential to counteract TGF-β–mediated suppression and restore granzyme B secretion in NK-92 cells (**Figure 6**). The NE7 treatment group (IL-15 + TGF-β + NE7) produced granzyme B levels statistically indistinguishable from the IL-15 only group (p = 0.46), suggesting that NE7 effectively mitigates the inhibitory effect of TGF-β. Statistically significant differences were observed between the control and NE7 group (p = 0.01), and between the IL-15 + TGF-β group and the IL-15 + TGF-β + NE7 group (p = 0.02).

### 3.10 *In vivo* human osteosarcoma xenograft model of pulmonary metastases

**Figure 7:**
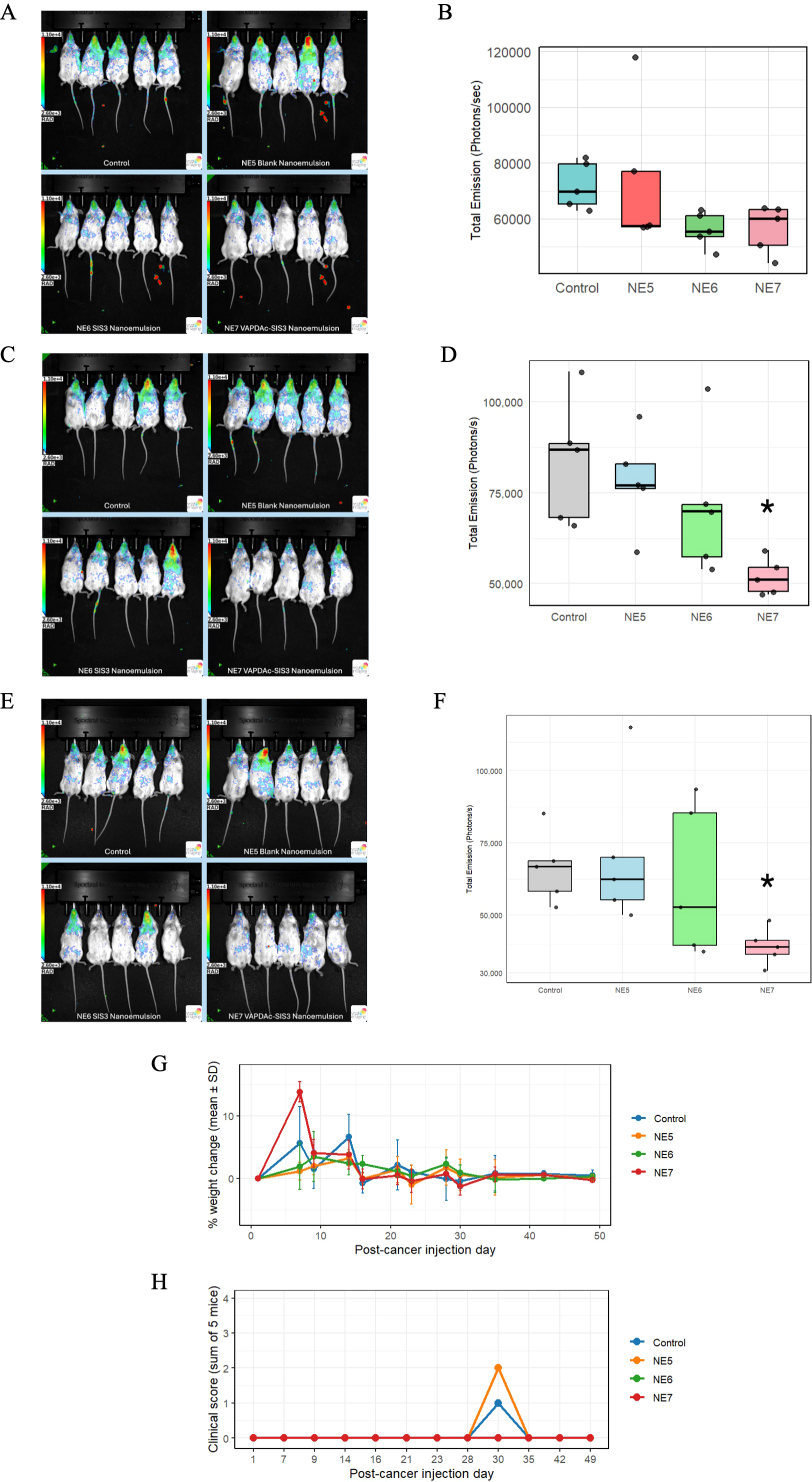
Combination SIS3-VAP–DAC nanoemulsion with adoptive NK-92 cell therapy reduces pulmonary osteosarcoma metastases *in vivo* without systemic toxicity. **(A)** Representative bioluminescence images of mice from each treatment group at week 1 of dosing. **(B)** Chest-region bioluminescence ROI values at week 1 across treatment groups. Box and whisker plots display quantitative ROI measurements from the chest area of individual mice, with the middle line indicating the median value. Data represent pulmonary tumor burden after one week of treatment. Whiskers represent data within 1.5 times the interquartile range. **(C)** Representative bioluminescence images of mice from each treatment group at week 2 of dosing. **(D)** Chest-region bioluminescence ROI values at week 2 across treatment groups. Box and whisker plots display quantitative ROI measurements from the chest area of individual mice, with the middle line indicating the median value. Data represent pulmonary tumor burden after two weeks of treatment. Whiskers represent data within 1.5 times the interquartile range. **(E)** Representative bioluminescence images of mice from each treatment group at week 3 of dosing. **(F)** Chest-region bioluminescence ROI values at week 3 across treatment groups. Box and whisker plots display quantitative ROI measurements from the chest area of individual mice, with the middle line indicating the median value. Data represent pulmonary tumor burden after three weeks of treatment. Whiskers represent data within 1.5 times the interquartile range. At weeks 2 and 3 **(D, F)**, asterisks denote statistically significant differences between NE7 and the control group by Kruskal–Wallis non−parametric analysis of variance followed by Dunn’s *post hoc* test (*: *p* < 0.05, Bonferroni corrected). No significant differences were detected at week 1 **(B)**. **(G)** Percentage change in mean body weight over time for control and nanoemulsion-treated mice (NE5-NE7) following MG63 cancer cell injection. Error bars represent ± SD of the percentage changes from baseline at each timepoint. **(H)** Summed clinical scores in control and nanoemulsion-treated mice (NE5-NE7) over 49 days after MG63 cancer cell injection. Points at zero indicate no observable adverse clinical signs.

While potential beneficial effects of the dual SIS3-VAP–DAC nanoemulsion NE7 could be observed *in vitro* on NK cells and on tumor cells, it was unclear if the dual nanoemulsion would have sufficient bioavailability to mediate similar benefits *in vivo*. NSG mice were injected with luciferase-expressing MG63 human osteosarcoma cells and then treated with weekly NK-92 intravenous infusions and intranasal nanoemulsion. By week 1 (**Figure 7A**), mice had received a total of two doses; by week 2 (**Figure 7C**), five doses; and by week 3, eight doses (**Figure 7E**). Across this timeline, mice treated with intravenous adoptive transfer of NK cells and intranasal NE7 consistently exhibited reduced bioluminescence signals in the lungs compared to the control group, indicating reduced tumor burden. Quantitative ROI measurements were measured, and no statistical difference was observed in week 1 (**Figure 7B**). Quantitative ROI measurement started to show a statistically significant difference between the control and NE7 groups beginning at week 2 (p < 0.05) (**Figure 7D**), which persisted through week 3 (**Figure 7F**). Body weight change in mice remained essentially stable in all groups. No significant loss in weight (**Figure 7G**) or meaningful rise in cumulative clinical scores (**Figure 7H**) was observed. Overall, adoptive NK-92 infusions combined with intranasal administration of NE7 resulted in decreased osteosarcoma metastatic burden in the lungs without noted systemic toxicity.

## 4 Discussion

Despite the severe impact of pulmonary metastases on patient prognosis and quality of life (46), no treatments are currently approved that specifically target pulmonary metastases (47). While there is promising preclinical data in mice (48–51), adoptive cell therapy with NK cells for treating metastatic osteosarcoma has had limited success to date in human and canine clinical trials (52, 53), implying novel approaches are sorely needed. Inhaled agents that can activate NK cells within the immunosuppressive pulmonary tumor microenvironment have been tested, but to date have involved a single agent aimed at activating NK cells like inhaled IL-2 (48), inhaled IL-12 gene therapy (54), inhaled IL-15 (55), and inhaled GM-CSF (56). None of these inhaled agents block inhibitory signals on NK cells mediated through non-overlapping pathways. This study aimed to develop a novel inhalable drug delivery system to enhance adoptive NK cell therapy against osteosarcoma pulmonary tumor burden by blocking 2 non-overlapping inhibitory signals - TGF-β signaling and ILT-2 binding to HLA-C.

Chronic exposure to TGF-β desensitizes NK-cell activation, downregulating NKG2D (57) and perforin/granzyme expression (58), thereby impairing tumor cytotoxicity. An inhalable oil-in-water nanoemulsion was loaded with the TGF-β inhibitor SIS3 and surface-decorated with the KIR antagonist VAP–DAC peptide, called SIS3-VAP–DAC, aiming to create a safe and effective strategy for treating pulmonary metastatic osteosarcoma. SIS3 was chosen for its ability to selectively inhibit SMAD3 phosphorylation in NK cells, thereby restoring NK-cell cytotoxic functions after exposure to TGF-β. The VAP–DAC peptide was included to antagonize the HLA-C/KIR or ILT-2 interactions responsible for tumor-induced suppression of NK-cell activity. To our knowledge, SIS3-VAP–DAC is the first drug to block both TGF-β signaling and KIR/ILT-2, and antagonism of both of these inhibitory pathways has not been evaluated simultaneously on NK cells in the setting of cancer therapy.

In order to optimize treatment of pulmonary metastatic tumors through intranasal delivery, we tested and optimized a DSG-PEG2000 surfactant nanoemulsion to verify its effects on nanoparticle size distribution, mucus turbidity and toxicity of small airway respiratory epithelium. We next incorporated the TGF-β inhibitor SIS3 within the nanoemulsion as a means of enhancing NK cell function from a contact-independent inhibitory signal. To further enhance the SIS3 concentration within the nanoemulsion, Plurol^®^ Diisostearique was incorporated as a co-surfactant, enhancing SIS3 solubility in the oil phase. We then added the KIR antagonist peptide VAP–DA onto the surface of the nanoemulsion to enhance NK cell function from the contact-dependent inhibitory signal from HLA-C. To enhance the ability of VAP–DA peptide to conjugate to PEG, which would enhance expression on the nanoemulsion, we added a cysteine residue (VAP–DAC) so that the nanoemulsion had functional surface expression of the VAP–DA peptide. We employed high-energy homogenization and microfluidization to formulate all nanoemulsions.

Adoptive cell therapy with irradiated NK-92 cells for cancer has already been examined in clinical trials (59–61), but with limited success in osteosarcoma (52). Drawbacks of NK-92 cells observed in clinical trials have included the lack of CD16 expression, limiting combination with antibody therapy, and need for irradiation prior to delivery, limiting *in vivo* expansion. *In vitro*, SIS3-VAP–DAC-modified nanoemulsions significantly enhanced NK92-mediated cytotoxicity against MG63 human osteosarcoma cells. Additionally, treatment of K7M2 murine osteosarcoma cells co-incubated with primary murine NK cells led to increased expression of RAE-1, an essential NKG2D ligand typically downregulated in TGF-β-rich tumor microenvironments. It is known that gamma interferon (IFNγ) stimulates RAE-1 expression, and since activated NK cells secrete high levels of IFNγ, it is possible that increased RAE-1 expression is a surrogate of NK activation by the nanoemulsion. ELISA assays further demonstrated that the SIS3-VAP–DAC nanoemulsion restored granzyme B secretion in TGF-β-suppressed NK-92 cells to levels comparable to IL-15 stimulation, with significant improvements observed versus untreated and TGF-β-only groups.

For *in vivo* testing, a human osteosarcoma xenograft mouse model with established lung metastases was used. Intranasal administration of the SIS3-VAP–DAC nanoemulsion over three weeks, combined with intravenous adoptive transfer of irradiated NK-92 cells, significantly reduced pulmonary tumor burden compared to controls, without adverse effects on body weight or clinical scores. Collectively, these findings demonstrated that the SIS3-VAP–DAC nanoemulsion is physically stable, biocompatible, and effectively reactivates NK-cell immune functions within a TGF-β-rich tumor microenvironment, significantly reducing pulmonary tumor burden. These data also indicate concurrent blockade of TGF-β/SMAD3 and ILT-2/HLA-C pathways may produce synergistic reactivation of NK-cell function by restoring both intracellular signaling and surface receptor responsiveness. A limitation of the experimental design was that the nanoemulsion was not tested without infusing NK-92 cells. Nevertheless, this inhalable formulation represents a promising non-invasive therapeutic strategy for enhancing adoptive NK cell therapy of metastatic osteosarcoma to the lung.

## 5 Conclusions

Future directions should further examine the impact of blocking TGF-β and KIRs on primary human NK cells following SIS3-VAP–DAC nanoemulsion treatment, which may offer deeper insights into the underlying immunomodulatory mechanisms and determine if blocking HLA-C interactions preferentially impacts inhibitory or activating KIRs. Combination treatment with adoptive NK cells or chimeric antigen receptor (CAR) NK cells could also be explored. *In vivo* studies could explore dual administration approaches, combining intranasal with systemic delivery, to potentially enhance therapeutic efficacy and more robustly suppress metastatic progression. Additionally, investigating the use of intranasal SIS3-VAP–DAC nanoemulsion as a safe and effective adjuvant to standard of care systemic chemotherapy could further improve outcomes by reducing pulmonary metastatic burden. Finally, future studies analyzing the nanoemulsion’s effects on other cell subsets within the tumor microenvironment could offer additional insights into its therapeutic potential with other adoptive cell therapies impacted by TGF-β, KIR and ILT-2 (e.g., NKT cells, γδ T cells, etc.).

## Data Availability

The original contributions presented in the study are included in the article/**Supplementary Material**. Further inquiries can be directed to the corresponding authors.
