# Supplementary material for: Development and evaluation of an inhalable nanoemulsion system for enhancing NK cell function against osteosarcoma pulmonary metastases
Source: Front Immunol. 2026 Feb 12;17:1772375. doi: 10.3389/fimmu.2026.1772375 (PMC12936002; doi:10.3389/fimmu.2026.1772375)
Supplement: Supplementary file 1 [file DataSheet1.docx]

Supplementary Material

# Supplementary Figures and Tables

## Supplementary Figure 1.

## Supplementary Figure 1: SIS3-VAP–DAC nanoemulsion enhances the percentage of murine osteosarcoma cells expressing the NKG2D ligand RAE-1. Analysis of RAE-1 expression on K7M2 murine osteosarcoma cells (NKp46^-^RAE-1^+^) following treatment with nanoemulsions (NE1-NE4).

##

## Supplementary Table 1.

## Supplementary Table 1: Specific ingredients and their concentrations used for the preparation of nanoemulsions.

| Nanoemulsion | DSG-PEG2000 | DSPE-PEG2000 | VAP–DAC-DSPE-PEG2000-Mal | Final SIS3 Conc. |
| --- | --- | --- | --- | --- |
| NE1 | 0.95 mM | 0.05 mM | N/A | N/A |
| NE2 | 0.95 mM | 0.05 mM | N/A | 124.92 μM |
| NE3 | 0.95 mM | N/A | 0.05 mM | 109.10 μM |
| NE4 | 0.95 mM | N/A | 0.05 mM | N/A |
| NE5 | 0.95 mM | 0.05 mM | N/A | N/A |
| NE6 | 0.95 mM | 0.05 mM | N/A | 240 μM |
| NE7 | 0.95 mM | N/A | 0.05 mM | 240 μM |

## Supplementary Table 2.

## Supplementary Table 2: Clinical scoring criteria used to assess mouse health during the dosing schedule.

| **Criteria** | **Grade 0** | **Grade 1** | **Grade 2** |
| --- | --- | --- | --- |
| Weight Loss | <10% | >10% to <25% | >25% |
| Posture | Normal | Hunching noted only at rest | Severe hunching impairs movement |
| Activity | Normal | Mild to moderately decreased | Stationary unless stimulated |
| Fur Texture | Normal | Mild to moderate ruffling | Severe ruffling or poor grooming |
| Skin Integrity | Normal | Scaling of paws or tail | Obvious areas of exposed skin |
